# Mottle: Accurate pairwise substitution distance at high divergence through the exploitation of short-read mappers and gradient descent

**DOI:** 10.1371/journal.pone.0298834

**Published:** 2024-03-21

**Authors:** Alisa Prusokiene, Neil Boonham, Adrian Fox, Thomas P. Howard

**Affiliations:** 1 Faculty of Science, Agriculture and Engineering, School of Natural and Environmental Sciences, Newcastle University, United Kingdom; 2 Fera Ltd., Biotech Campus, York, United Kingdom; Bristol-Myers Squibb Company, UNITED STATES

## Abstract

Current tools for estimating the substitution distance between two related sequences struggle to remain accurate at a high divergence. Difficulties at distant homologies, such as false seeding and over-alignment, create a high barrier for the development of a stable estimator. This is especially true for viral genomes, which carry a high rate of mutation, small size, and sparse taxonomy. Developing an accurate substitution distance measure would help to elucidate the relationship between highly divergent sequences, interrogate their evolutionary history, and better facilitate the discovery of new viral genomes. To tackle these problems, we propose an approach that uses short-read mappers to create whole-genome maps, and gradient descent to isolate the homologous fraction and calculate the final distance value. We implement this approach as *Mottle*. With the use of simulated and biological sequences, *Mottle* was able to remain stable to 0.66–0.96 substitutions per base pair and identify viral outgroup genomes with 95% accuracy at the family-order level. Our results indicate that *Mottle* performs as well as existing programs in identifying taxonomic relationships, with more accurate numerical estimation of genomic distance over greater divergences. By contrast, one limitation is a reduced numerical accuracy at low divergences, and on genomes where insertions and deletions are uncommon, when compared to alternative approaches. We propose that *Mottle* may therefore be of particular interest in the study of viruses, viral relationships, and notably for viral discovery platforms, helping in benchmarking of homology search tools and defining the limits of taxonomic classification methods. The code for Mottle is available at https://github.com/tphoward/Mottle_Repo.

## Introduction

Pairwise nucleotide substitution distance is widely used in bioinformatic analyses. Pairwise comparisons within collections of genomes are commonly integrated to establish phylogenies, providing insight into their shared evolutionary history. They are similarly used to position novel genomes within an established phylogeny [1]. Substitution distances can also be converted to genome-wide percentage identity to define taxonomic demarcations, such as the distance a novel genome must be from known genomes to establish it as a new species [2]. Comparing the distances of discrete genetic molecules such as chromosomes, plasmids, plastids, and segments can find differences in their evolutionary histories, elucidating how genetic material has been exchanged between organisms or populations. Finding the most distinct or representative sequences in a set is used to create a compressed database for homology search and taxonomic classification [3]. A high substitution distance can however be a large barrier for sequence discovery [4]. Being able to accurately define this distance allows improved benchmarking of homology search and taxonomic classification tools to find their limits, allowing accurate estimates of what may pass through the silicon sieve of *de novo* sequencing-based discovery and diagnostics.

Despite the considerable number of approaches available for pairwise genome comparison [5], many do not produce a biologically relevant substitution distance, and from those that do, there are few that are suitable for highly divergent sequences, i.e., those that have had many substitutions per site between them. This is for two main reasons–work has focused on creating faster and more efficient tools that work well at low divergences [6–11], combined with the inherent difficulties found when making comparisons at high divergences. The largest of these difficulties are false seeding and over-alignment. Seeding is the process of finding small sub-sequences, often in the tens of bases, that are identical or near identical in two sequences and can be extended to larger regions of homology. Finding seeds at non-homologous locations, i.e., false seeding, may add regions that otherwise have little similarity into the distance calculation, artificially inflating it. At low divergences a large seed size may be used as there would be few mutations between homologous regions. At increasing divergence, a smaller seed size is needed to find such regions, generating many seeding locations that are spurious, eventually overwhelming the limited number of truly homologous seed sites. Finding seed locations that have true homology while avoiding or removing false ones becomes a critical task at distant homologies. While false seeding can make sequences appear more divergent than they truly are, over-alignment can make them appear more similar. Alignment is usually done after seeding, inserting gaps into either sequence to match up homologous nucleotides that were shifted due to insertions and deletions (indels). This presents the danger of over-correcting for indels, falsely pairing matching nucleotides that are non-homologous, such as mistaking adjacent substitution events for an insertion, and therefore not contributing to the substitution distance. Avoiding over-alignment while still correctly aligning regions can be difficult at a low divergence, but at a high divergence, where multiple different mutation events may have occurred at the same nucleotide position, it may not be possible.

These difficulties appear especially often when studying viral genomes, due to their high rate of mutation, small size, lack of universal marker genes, and the low proportion of known viruses [12,13]. Viral replication machinery, especially those of single-stranded RNA based viruses, are known to introduce many substitutions every generation. Combined with a short generation time, these viruses can quickly diverge from their progenitor genome. Additionally, the ratio of indel event to substitution events is extremely high in these genomes [14], which makes finding true seeds more difficult and over-alignment more likely. The usually small size of viral genomes further reduces the number of possible seeding sites. Finally, the number of viral species that have been documented is a small proportion of the total estimated number of viral species [15], with estimates of the proportion of characterised orthornaviran RNA viruses being estimated as low as 0.006% [16], and environmental sampling projects discovering many previously unobserved viral genomes with each sequencing experiment [17]. The effect of sparse taxonomic coverage is that many viral genomes are highly diverged from any known viruses, making it difficult to study their relationship to other viruses. To this end, viral genome analysis is the field that may benefit the most from improvements in pairwise sequence distance accuracy at high divergences.

In the face of these difficulties, current tools attempt to alleviate parts of the false seeding or over-alignment problems: some programs attempt to find non-exact matches for seeding, finding longer seeds which are more likely to be true seeds [18]. Others, such as Hachiya et al. [19], have sophisticated algorithms for distinguishing between true and false seeds. Programs that do not use any alignment step, known as alignment-free programs, are able to completely avoid the problem of over-alignment, instead using statistical information from across a sequence that is correlated to substitution distance–e.g., proportion of shared k-mers [20], shortest unique substrings [21], average common substring [22], or sequence embeddings [23]. This creates a separate issue–as sequence divergences increase, the correlation between these global statistical data and the local nucleotide substitutions they estimate can become increasingly decoupled. A new approach is needed, one which creates high-quality seeds and avoids over-alignment while incorporating direct nucleotide-level information.

Short-read mappers (henceforth referred to as mappers) are tools used to map short sequence fragments of ~50-300bp, produced from High Throughput Sequencing runs, to reference genomes. These mappers already employ sophisticated seeding algorithms, give many parameters to tune mapping sensitivity, can use arbitrary queries and references, and are designed to handle thousands to millions of fragments. Sequence fragments created *in silico* can therefore be used as inputs to these mappers, finding the optimal homologous location for each fragment on another sequence, and allowing downstream processing to calculate substitution distance. This approach of running mappers on *in silico* fragments has been successfully utilised in other bioinformatic applications–for generating multiple sequence alignments in *ViralMSA* [24], and for constructing phylogenies in *REALPHY* [25]. In this paper we explore the application of these mappers for estimating pairwise nucleotide substitution distance, how careful use of their outputs can avoid false seeding and over-alignment and describe the implementation of *Mottle*—a tool for more accurate distance calculation between two highly divergent sequences. The tests carried out within this paper are based on consensus sequences of full viral genomes or RNA family sequences, but Mottle can be applied to any pair of DNA or RNA sequence files to give an estimated substitution distance.

## Design and implementation

*Mottle* takes two arbitrary nucleotide sequences of unknown relation, and outputs an estimated substitution distance between them. This can make use of any mapping software that aligns short fragments to larger sequences. Additionally, we have implemented a bespoke fragment mapping algorithm for this process, *Mottle-map*, which guarantees that each fragment is mapped but is not scalable to large sequences. Both algorithms are described in the subsections below.

### Mottle: Calculating pairwise sequence distance from mapped fragments

**Fig 1:**
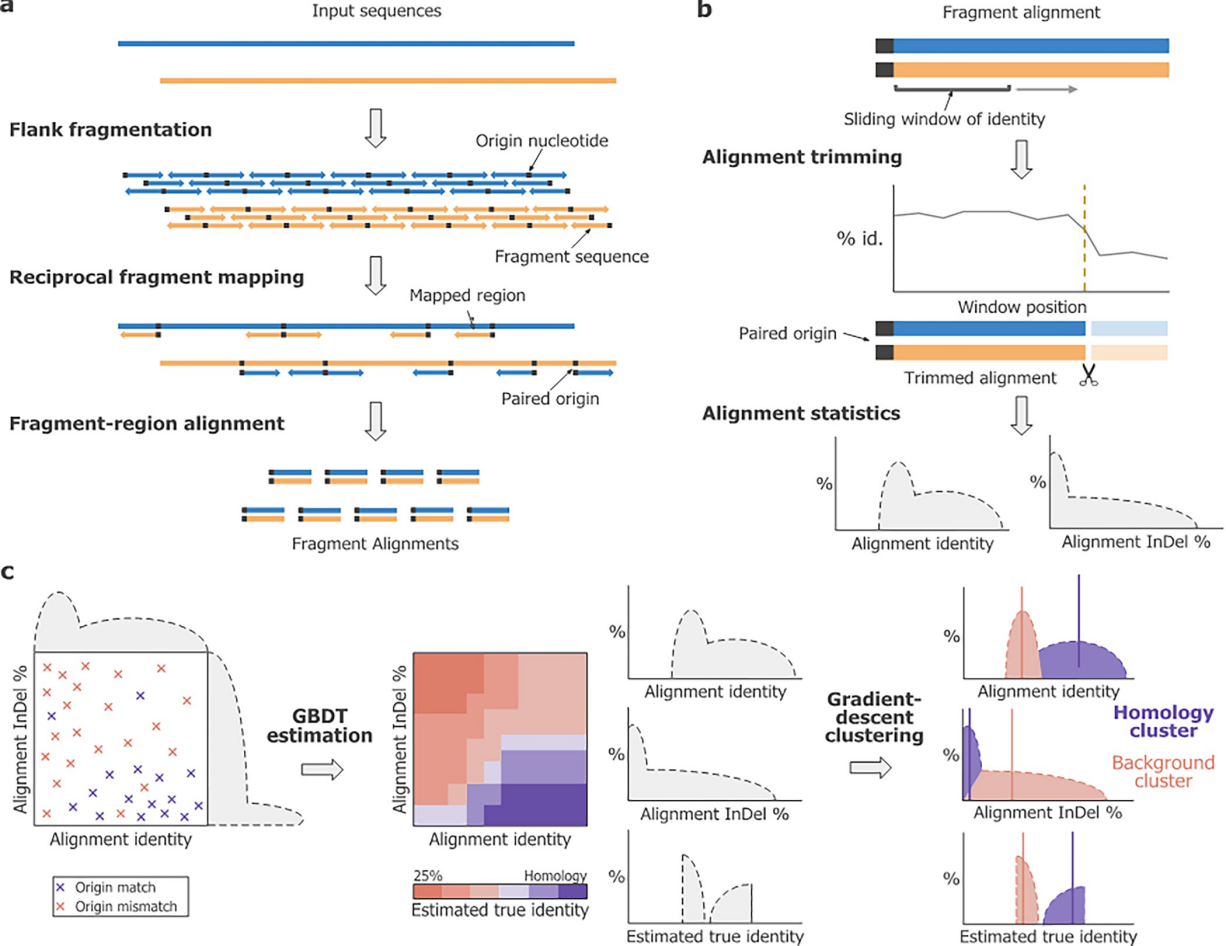
Overview of *Mottle*’s sequence distance estimation algorithm. **(a)** Generating fragment alignments from input sequences. Each sequence is fragmented *in silico*. The origin nucleotide is excluded from each fragment sequence. Fragments are mapped onto the reciprocal sequence via a mapper, with each mapped fragment’s origin being paired. Origin pairs carry a binary state (match or mismatch). Fragment sequences are then fully aligned. **(b) Truncating** alignments on identity change. For each alignment, a sliding window calculates percentage identity. If a window’s identity diverges from the initial window’s, all nucleotides from that point onwards are discarded. **(c)** Fragment clustering and substitution distance estimation. For each alignment, identity and indel percentage statistics are calculated. These are fed into a Gradient Boosted Decision Tree (GBDT), which is trained to predict origin pair match state. This gives a predicted match probability on each alignment that can be interpreted as a bias-free identity. These three statistics are used for gradient-descent clustering, to find a cluster of alignments that were generated due to shared homology, and a cluster for those due to chance. Once both fractions are obtained, a mean origin identity is calculated for the homology cluster, which is used to derive the final substitution distance between the two sequences.

The main *Mottle* program can be split into three stages–fragment generation, alignment processing, and alignment clustering, presented in Fig 1A to c respectively and described in the following sections. Briefly, for each position, *p*, in query genome, Q, we set the nucleotide at that position, Q_*p*_, as the origin nucleotide. The set of flanking regions either side, Q_[*p+*1..*p*+n]_ and Q_[*p-1*..*p*-n_] for a specified flank size *n*, of each origin nucleotide are mapped using a short-read mapper to a similar set of flanking regions in the target genome. For each mapping, we calculate flank alignments, and gather a set of statistics: the alignment identity, the indel rate of the alignment, and a binary value representing whether the corresponding origin nucleotides match or mismatch. We represent mappings as points defined by identity and indel statistics, labelled by match state, from which we can calculate a match probability that acts as an estimator for true identity without alignment bias. These points are clustered into two sets to separate homologous mappings from spurious ones, and genomic distance is estimated from the homologous set.

#### (a) Fragment generation

*Flank fragmentation*. Each sequence can be separated into a discrete set of subsequences, each defined by a unique combination of origin nucleotide, subsequence size, and relative orientation. Each nucleotide in the full-length sequence can act as an origin—the first nucleotide of a subsequence—of multiple subsequences. But the possible sizes and relative orientations of these subsequences are restricted depending on the origin nucleotide’s location. If subsequence size is kept fixed, each nucleotide is an origin for up to two subsequences, for each of the forward and reverse complement orientations. The portion of each subsequence that does not contain the origin nucleotide is termed a fragment, and the two possible fragments for each origin are termed the forward and reverse flank fragments of the origin. Considering the origin nucleotide separately from the fragments is integral in estimating true identity in the later stages of the algorithm.

*Reciprocal fragment mapping*. This step takes the two full-length input sequences, their generated fragments, and a mapper program, to create a mapping between each fragment and a homologous area in the sequence of comparison. To allow the use of a variety of mapping algorithms, there are multiple modes of input depending on what the mapper accepts–sequence and fragments, fragments and fragments, or sequence and sequence. The mappings are done reciprocally–one sequence is used as the query with the other acting as the reference, and vice versa. The output of a mapper is a series of possibly homologous regions between the two inputs. This must either contain fragment names or sequence locations to allow identification of origin nucleotide, query location, and mapped location for each region. In cases where a mapper returns multiple regions for a query, all mapping is kept. The mapped region sequence pairs and corresponding origin nucleotides are extracted for use in later steps.

*Mapped region alignment*. While some mappers output a full alignment between mapped regions, many do not. To get consistent and well-defined alignments, Needleman-Wunsch global alignment is carried out between region sequences independent of mapper. This is because a representative region of homology adjacent to the origin nucleotide is desired, which Needleman-Wunsch global alignment maximises. Aligned regions that contain gaps in the first N bases are discarded, where N is a parameter of *Mottle*, as these gaps may shift the aligned region of homology and therefore the origin nucleotide.

#### (b) Alignment processing

*Alignment truncation*. Not all alignments will contain a consistent homology throughout. Some may begin with a high degree of similarity, but with a genomic rearrangement or large indel creating a discontinuity that suddenly reduces similarity. This non-homologous section would change alignment properties, adding noise to statistics calculations, and confounding downstream clustering. To remove these discontinuities, a sliding window is moved through the alignment. The length and identity, the proportion of aligned nucleotides that match, of the first window is used to estimate a binomial distribution for match/mismatch states. Where a later window’s identity is above or below that which would be expected by chance of this distribution, the alignment is clipped, discarding proceeding nucleotides. Clipped alignment shorter than a minimum size are additionally discarded. The window size, two-tailed binomial test p-value, and minimum clipped alignment size are configurable parameters.

*Alignment statistics calculation*. To distinguish homologous from non-homologous alignments, a set of statistics is calculated for each of the non-discarded windows produced during truncation. Alignment identity is the fraction of matches in non-gap positions, corrected for the GC composition of the alignment. The fraction of gap positions in the window could be used as another such statistic, where homologous alignments would contain fewer gaps at the same identity. This would, though, not inform us of the probability that the origin nucleotide is shifted by an Indel event, as each event can insert or delete multiple nucleotides in a row. The number of Indel events in a row between non-gap positions can be approximated by a geometric distribution with PMF,

$$P(L=l)=p^{l}\cdot (1-p)$$

where *l* is the number of adjacent indel events between two non-gap positions regardless of the size of each event, *P*(*L = l*) is the probability of finding this number of events between two arbitrary sites in the sequence, and *p* is the fraction of indel events per site. *p* is directly related to the probability of no indel events having occurred between two adjacent homologous nucleotides via *P*(*L* = 0) = 1—*p*, which can be estimated from the fraction of adjacent aligned nucleotides that do not have gaps, *q*, in an observed sequence, (*p* = 1 –*q*). This produces a set of identities and indel fractions for each alignment—one for every fixed-size window within it. After this step, the alignments themselves are discarded, with only these statistics and the origin nucleotide match state being kept for the final steps.

#### (c) Alignment clustering

*GBDT estimation of true identity*. Identity and indel fraction can greatly vary between and within homologous alignments. For clustering to be effective, a more stable statistic needs to be calculated. For this purpose, we train a Gradient-Boosted Decision Tree (GBDT) to estimate a ’true identity’ for each window over the space defined by the previous statistics (Fig 1C, ’GBDT estimation’). This treats each window as a single point of data, with parameters identity and indel fraction, and with prediction value being origin nucleotide match state (1 = match, 0 = mismatch). The output of a GBDT can be thought of as a stepwise surface embedded within the input parameter space, which we enforce to be monotonically increasing with window identity and decreasing with indel fraction. This estimates the proportion of origin nucleotides that match within each area of parameter combinations, which we term the true identity estimate. As a wide area can have the same value estimated, similar alignments are likely to have windows that share similar true identities, making clustering more stable.

*Gradient-descent clustering*. The final step in *Mottle* is to isolate the homologous fraction of alignments through clustering. This approach involves the estimation of two cluster centres, one for homologous alignments (homologous cluster) and one for non-homologous alignments (null cluster), within the three-dimensional space defined by Alignment Identity, indel Fraction, and True Identity statistics. For every alignment, a set of windowed statistics had been generated from during alignment processing, meaning that each alignment contains a distribution of these statistics. Similar to how we approximated runs of indel events with a geometric distributions, we approximate these alignment statistics with a Binomial distribution. Clustering using these statistics directly would be difficult, as the expected variance of Binomially distributed values greatly decreases when the probability approaches zero or one. To rectify this, we utilise the following variance stabilising transformation,

$$\frac{\sin^{-1}\left(2\cdot \frac{\max\left(p,l\right)-l}{1-l}-1\right)}{2\pi }$$

where p is equal to the value of the statistic, and l is equal to the expected lower bound of the statistic, i.e. 0.25 for GC-corrected nucleic acid sequences and 0 for indels. We then define a normalised distance to each cluster centre as,

$$dist=\sqrt{{\left(\frac{{mean}_{I}}{{var}_{I}}\right)}^{2}+{\left(\frac{{mean}_{D}}{{var}_{D}}\right)}^{2}+{\left(\frac{{mean}_{T}}{{var}_{T}}\right)}^{2}}$$

With *mean*_*I*_ and *var*_*I*_ equal to the mean and variance of the windowed set of stabilised alignment identities. Similarly, *mean*_*D*_ and *var*_*D*_ are generated from the set of indel fractions and *mean*_*T*_ and *var*_*T*_ from estimated true identities. The centre of the null cluster is initialised so that its True Identity is equal to the expected proportion of matches if it was due to chance, i.e., -1 after the stabilising transformation. To initialise the Alignment Identity and indel fraction, we choose the values of the alignment with the mean True Identity closest to this value. The centre of the Homologous cluster is initialised to the values of the alignment at the 90^th^ percentile of mean True Identities. Once initialised, the cluster centres are shifted via gradient descent, with exception of the True identity of the null cluster which always remains at the expected null value, to reduce normalised distances between alignments and centres. The loss function for this process is as follows,

$$loss=\frac{\sum_{i=0}^{N}\sum_{j=0}^{S}\left(dist_{ij}\cdot weight_{ij}\right)}{\sum_{i=0}^{N}\sum_{j=0}^{S}\left(weight_{ij}\right)}$$

where dist is equal to the distance between the alignment *i* and the cluster centre *j*, N is equal to the total number of alignments, and S is equal to the number of clusters, which is set to two corresponding to the null and homologous clusters. Additionally, weight is defined as follows.

$$weight={\left(\frac{1}{\max\left(dist,eps\right)}\right)}^{\frac{1}{binpow}}$$

where binpow and eps are program parameters. A BFGS optimiser [30] is used to find the locations that give the minimum loss values. Alignments are then each assigned to the cluster with the closest centre. The final distance value, *D* returned by *Mottle* is obtained from the origin match proportion of alignments in the homology cluster, corrected for GC-content, and transformed to an estimate of substitution distance via the Jukes-Cantor (JC) model [26],

$$D=-\frac{3}{4}ln\left(1-\frac{4}{3}(1-I)\right)$$

With *I* being equal to mean true identity of the homologous cluster.

### Mottle-map: Bespoke fragment mapping algorithm

While *Mottle* may use any short-read mapper for fragment mapping, a bespoke algorithm was developed to ensure that each position in a sequence is mapped even at large divergences. *Mottle-map* achieves this by transforming each fragment to a high-dimensional embedding via the Fast Fourier Transform (FFT) and subsequently finding the nearest neighbour in the reciprocal sequence. This is similar to the approach *Satsuma* takes for synteny detection [27]. To allow the FFT transformation of fragments, their values must first be mapped to the complex number plane. For this we use the same embeddings as MAFFT [28], with each base placed at axis-aligned unit lengths and bonding pairs placed in opposite sides of the origin G → + 1, C → - 1, A → + i, T/U → - i. If each position in the fragment is treated as an embedding dimension, then two sequences that are more similar will have a smaller Euclidean distance between embedding, if there are no indels, that give a mid-sequence shift that would misalign the embedding. Shifting this embedding into frequency space via the FFT allows a Euclidean distance calculation while allowing for indels. Before transformation, we divide the real and imaginary axes by the mean of their absolute values, to correct for GC-content. Afterwards, the frequency embeddings are normalised to unit L_2_-norm. These steps can be represented mathematically as follows,

$$corrected=\frac{Re[E]\cdot L}{\sum_{i=0}^{L}Re[E_{i}]}+i\cdot \frac{Im[E]\cdot L}{\sum_{i=0}^{L}Im[E_{i}]}$$

where, E is the set of embedded nucleotides of a sequence, and L is the length of the sequence, and

$$normed=\frac{F}{\sqrt{\sum_{i=0}^{L}F_{i}^{2}}}$$

Where F is the set of values generated from GC-corrected embeddings after transformation by FFT, and L is the length of the transformed sequence. Since Euclidean distance between frequency embeddings can be used as a dissimilarity metric between fragments, a nearest-neighbour search can be used between two sets of embeddings to find similar fragments. Since *Mottle-map* is made for small and highly-divergent sequences, we use a simple many-to-many comparison where the distance between each embedding vector is computed. The top N nearest-neighbour mappings for each fragment in two fragment sets are returned by *Mottle-map*, where N is a parameter.

### Implementation and availability

*Mottle* and *Mottle-map* are implemented in Python 3.9 and both are available at https://github.com/tphoward/Mottle\_Repo. Gradient-Boosted Decision Trees are calculated by LightGBM [29], gradient-descent clustering by Tensorflow-probability [30], and nearest neighbour search by Faiss [31]. Parameters used within testing were as follows, ntrees: 100, nleaves: 32, learn_rate: 0.1, subsamp: 1.0, binpow: 64, learn_mult: 0.001, reltol: 1.00E-20, maxiter: 100, binthres: 0.75, and prior_size: 10.

## Results and discussion

To evaluate the performance of *Mottle*, we run a set of benchmarks where exact or approximate relationships between sequences are known. We run *Mottle* with either *Mottle-map* or *BWA-MEM2* as the mapper. As a comparison, we include four other programs that are used for substitution distance calculation—*Co-phylog* which utilises micro-alignments between sequences [32], *Mash* which calculates distances based on shared k-mers [33], *Slope-SpaM* that uses spaced-word matches [34], and *Swipe* that calculates Smith-Waterman local alignments between sequences, utilising scores based on Karlin-Altschul statistics [35]. For the programs that output identities or alignments, a JC model is used to convert to substitution distances. A summary of the results of all benchmark tests can be seen in Table 1.

**Table 1:** Summary of benchmarking results.

Tool Name	Sub	Rfam	Gen-fam	Fam-ord	Ord-class
Slope-spam	0.52	0.22	0.92	0.70	0.37
Co-Phylog	0.28	0.12	0.82	0.38	0.12
Mash	0.44	0.18	**0.98**	0.85	**0.70**
Swipe	0.36	0.28	0.89	0.69	0.33
BWA-MEM2*	0.92	0.24	0.94	0.71	0.61
Mottle(-map)	**0.96**	**0.66**	0.95	**0.95**	0.75

Sub: Maximum stable distance of each program when tested on a simple *in silico* sequence evolution benchmark (section, *Simple sequence evolution*). Rfam: Maximum stable distance on concatenated RNA family alignments (section, *Known family alignments*). Gen-fam, Fam-ord and Ord-class: Proportion of correctly assigned outgroup genomes when comparing genomes in the same Genus/Family, Family/Order and Order/Class respectively (section, *Known genome taxonomies*). Scores in bold represent the best performing program in a benchmark test. *Here, *Mottle* was executed with BWA-MEM2 as the mapping software.

### Simple sequence evolution

**Fig 2:**
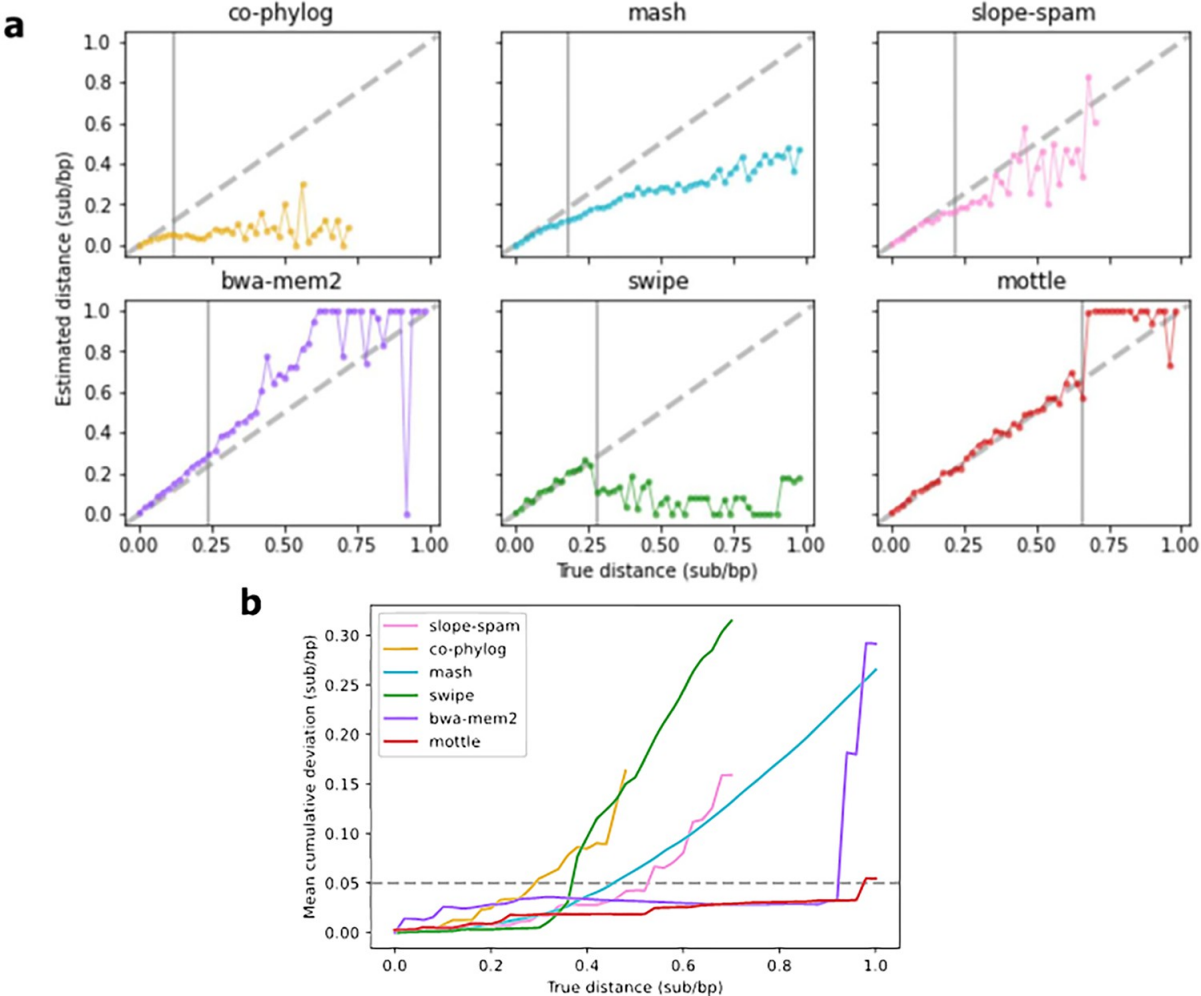
Accuracy of substitution distance prediction tools on a simple *in silico* substitution model of sequence evolution. **(a)** Program predictions vs true distance between sequences. Values are clipped to the range [0,1]. Vertical lines indicate the maximum stable distance. **(b)** Mean value of the cumulative deviation of each tool from the true distance. The maximum tolerable deviation is set to 0.05 sub/bp. The point at which curves cross tolerable levels defines the maximum stable distance. Curves are cut whenever NaN values are produced.

Our first goal was to test how well *Mottle* performed in comparison to other programs designed to calculate pairwise substitution distances, in the absence of confounding factors. Specifically, we tested programs against the Tobacco mosaic virus genome, with several substitutions introduced *in silico* to a set substitution distance and monitored their effectiveness at estimating this distance over increasing divergence. To do this, we used the genomic sequence of Tobacco mosaic virus (RefSeq accession NC_001367.1), and introduced a set number, *N*_*sub*_, of random substitution events *in silico*, following *N*_*sub*_ = *N*_*nuc*_⋅*D*_*sub*_, with *N*_*nuc*_ being equal to the size of the full sequence and *D*_*sub*_ being the desired substitution distance. Multiple substitution events were allowed to occur at each site. We then ran each substitution distance program on the original and modified genomes to find a calculated distance. This was run for every 0.02 substitution per base-pair (sub/bp) distance between 0 and 1 inclusive, giving a total sample size of 51 sequences. To have a measure of when the outputs of a program begin to consistently diverge from the true distance, that is independent of the number of trials, we calculated a running Mean Absolute Error (MAE) from the true distance as,

$$error=\sum_{K=0}^{N}\frac{\left|T_{k}-P_{k}\right|}{N}$$

where *error* is the calculated MAE, *T*_*k*_ being equal to the target substitution distance of a specified trial, *P*_*k*_ the output of a substitution distance program within this trial, and *N* increasing with the number of trials completed. As a threshold, we use an MAE value of 0.05 sub/bp, where the furthest distance a program’s output is within the MAE threshold is its maximum stable distance. The results indicated that many of the tools struggled to calculate the true distance between 0.25 and 0.35 substitutions per nucleotide base (Fig 2A, Table 1). Erratic behaviour was seen in many as they passed a critical threshold, set at 5% MAE from the true identity (Fig 2B). *Swipe* and *Mash* however tended to significantly underestimate true distance past this point. Interestingly, the results for *Mash* were incredibly stable after much deviation from the true distance (Fig 2A). The results indicated that *Mottle*, implemented with either *Mottle-map* or *BWA-MEM2* as the mapper, was able to accurately calculate the true distance over nearly all the sequence divergences tested, only reaching the 5% threshold towards the upper end of the testing space (Fig 2B, Table 1). Noticeable but non-critical deviations were observed in *BWA-MEM2* and *Mottle* throughout the testing space (Fig 2), such that a stricter threshold would have been surpassed at a lower divergence. The results for existing tools were surprising, and it was expected that they would perform reliably over a greater distance. It should be noted, however, that the divergence tested here (up to 1 substitution per base) would be considered high for many non-viral scenarios. These programs were not designed and tested with the high mutation rates observed in viruses in mind.

### Known family alignments

Having established that *Mottle* could successfully be used to predict true distance over a large sequence divergence space–albeit in a simplified system—we next wanted to test *Mottle* against real viral sequences. However, it was important to maintain knowledge of the true distance between sequences. A database that allows us to do this is the RNA families (Rfam) database [36]. Rfam catalogues homologous RNA sequences as families and holds multiple sequence alignments of them. In addition, while a simple substitution model can give an upper bound on the maximum stable distance for each program, indels are a common feature in biological sequences, especially those of viruses. To test how well *Mottle* can handle indels, we endeavoured to create a benchmark that incorporates *in vivo* substitutions and indels, while allowing us to know the ground truth in terms of substitution distance and giving a large range of such distances. This benchmark therefore assesses *Mottle* against both real viral sequences and sequences containing indels. To do this, we extracted all pairwise alignments in each family where both sequences are of viral origin and calculated a JC distance from their identities. To increase the length of these alignments and make them more comparable to a small RNA viral genome, alignments of similar divergence were concatenated until they reach the size of a small viral genome (>4 kb). For a given target distance, we find the alignment with the closest JC distance. We then calculate the current JC distance of the artificial genome, if below the target distance we add the closest alignment that is above the target distance and vice versa. This process is repeated without replacement until either the target length is reached, or we run out of valid candidate alignments. This created a more realistic dataset to test the six programs against. We run this benchmark on the same distances as previously (Simple sequence evolution, a total of 51 pairs of sequences with increasing substitution distance) and calculate MAEs in the same process.

**Fig 3:**
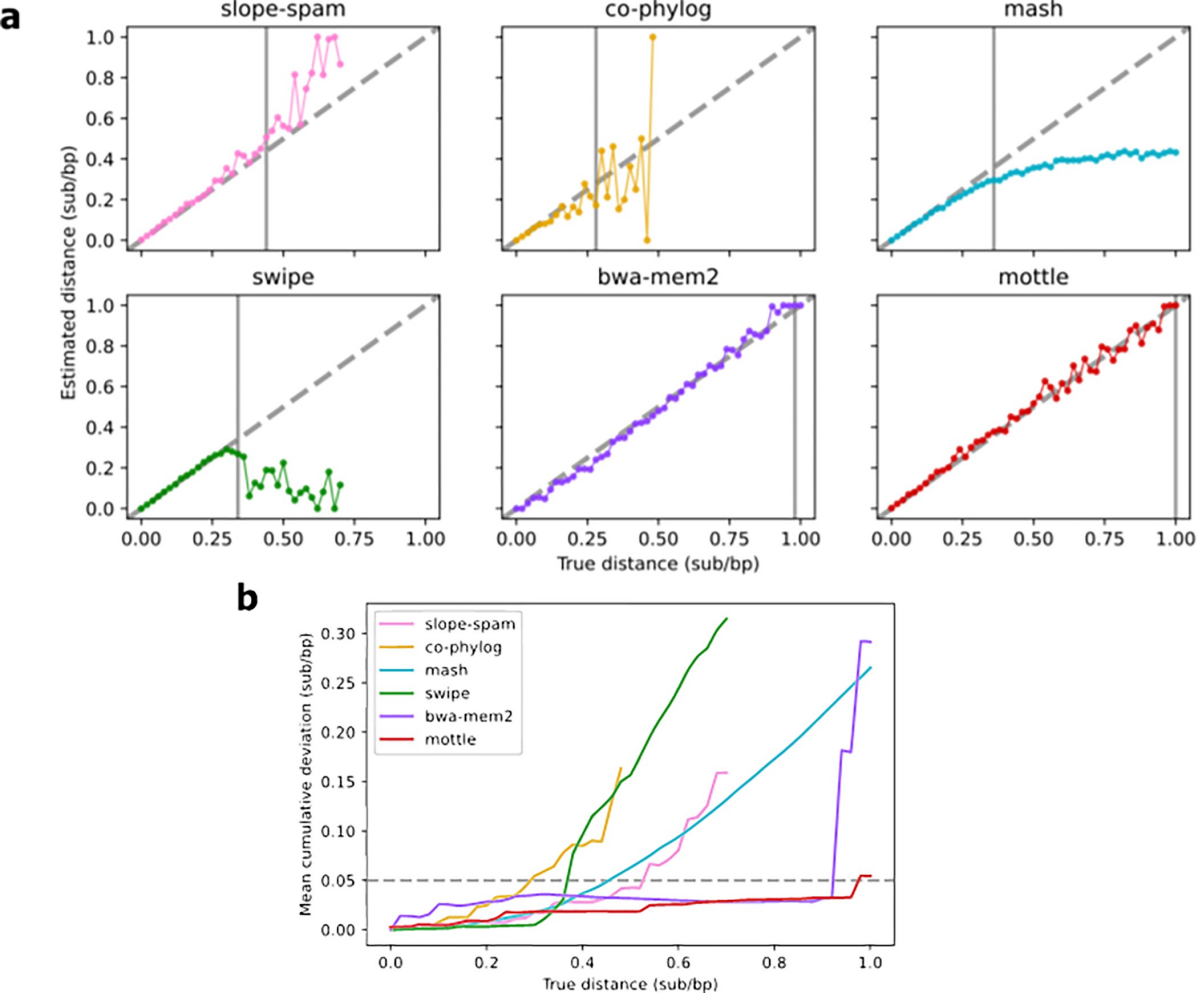
Accuracy of substitution distance prediction tools on a concatenated family alignment dataset. Formatted as Fig 2. **(a)** Program predictions vs true distance between sequences. **(b)** Mean cumulative deviation of each tool from the true distance.

For all tools, predictive performance was degraded compared to the previous test (Fig 3). For many programs, calculating distance was effective initially, but soon diverged from the true value. For most, distance was difficult to calculate beyond 0.25 substitutions per base (Fig 3A). *Co-Phylog*, *Swipe* and *Mash* tended towards underestimating sequence divergence from the true distance past this point, while *Slope-Spam* and *BWA-MEM2* displayed more erratic behaviour. By contrast, *Mottle* was able to track the true distance for longer, deviating only when reaching approximately 0.66 substitutions per base. All programs, except *Mottle*, had crossed the 5% cumulative deviation threshold by 0.3 substitutions per base (Fig 3B, Table 1). In contrast to Fig 2, all programs displayed similar deviation at low divergences. Taken together with the previous test, we conclude that *Mottle* is an effective tool for predicting true distance between highly divergent sequences, as may be found within viral populations.

### Known genome taxonomies

**Fig 4:**
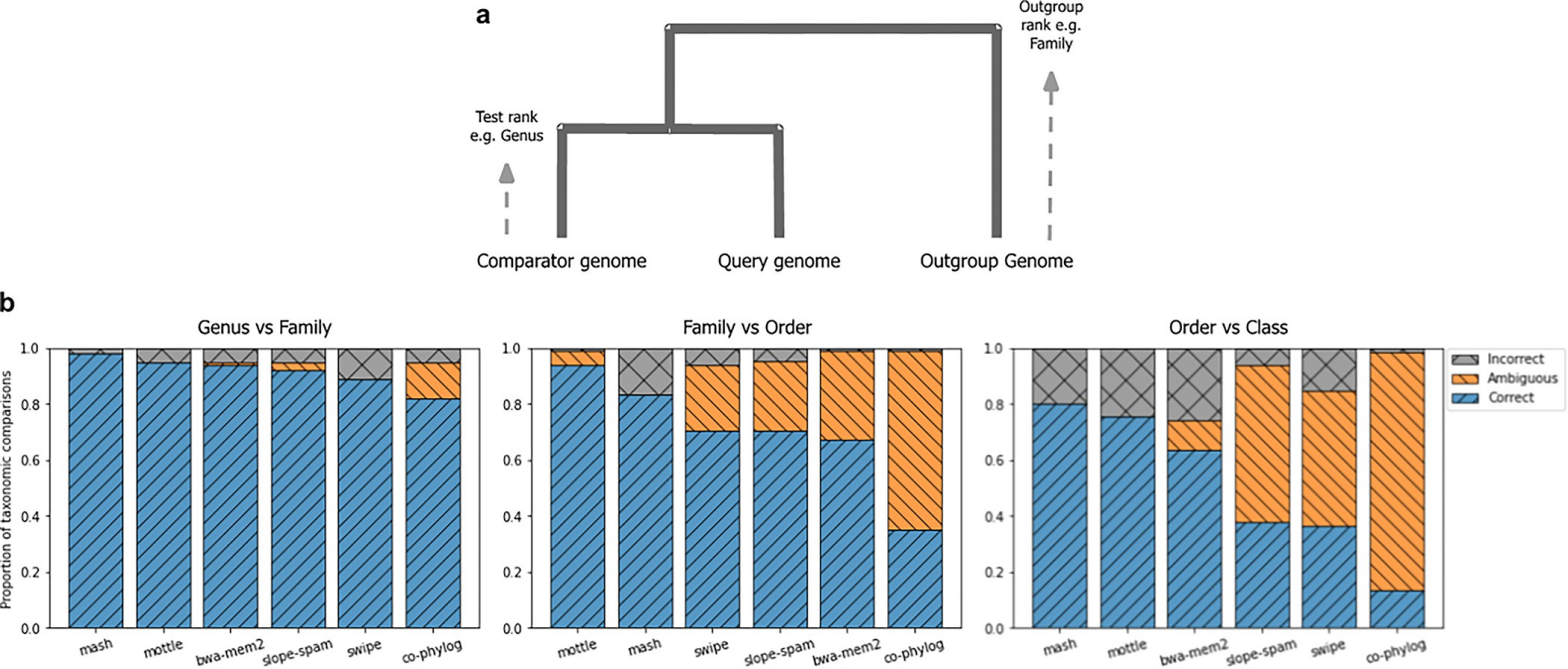
Accuracy of substitution distance prediction tools for identifying taxonomic outgroup genomes. **(a)** Taxonomic relationship between query, comparator and outgroup genomes. **(b)** Proportion of assignments that were correct (outgroup more distant than comparator genome), incorrect (outgroup less distant), or ambiguous (identical distances) for each tool when comparing genomes in the same Genus or same Family, in the same Family or same Order, and in the same Order or same Class, respectively.

We next wished to test the performance of these programs in identifying the relationship between genomes in the known viral taxonomy. We assembled a test set of viral genomes composed of three different genomes at two different taxonomic ranks (i.e., genus, order, family, class), as defined by the International Committee on Taxonomy of Viruses [37]. We used this dataset to assess how well each tool could identify outgroup genomes as the taxonomic rankings were increased. Briefly, each program was required to calculate the distance between one reference genome and two others, one that shares a chosen taxonomic ranking (comparator) and another that only shares the rank above (outgroup, Fig 4A). A correct identification gives the outgroup a higher distance to the reference. To carry out this benchmark, we select reference, comparator, and outgroup genomes randomly from the e NCBI taxonomy database [38] that exhibit this rank structure. On these, we run each program twice—once to calculate reference/comparator distance and once for reference/outgroup. If the distance to the outgroup is larger than to the comparator, this is recorded as a correct output, if smaller then incorrect, and if the output is the same for both then it is recorded as ambiguous. Some programs return NaN values or error codes if they are unable to find any homologous sites to calculate a distance from. In this case, the values are recorded as the maximal distance, which we store as infinity, and carry out the comparisons as before. The rank comparisons we used were Genus-Family, Family-Order, Order-Class. A total of 100 pairs of sequences were used within each rank of comparison.

Unsurprisingly, most of the programs were able to identify which genome shared the same Genus as the other, and which were only in the same Family (Fig 4B). *Mash* performed the best under these conditions, followed by *Mottle* and then *BWA-MEM2*. *Co-Phylog*, which was created with the genomes of cellular organisms in mind, did return several ambiguous results, perhaps a result of the instability of the output, even at low divergence, as seen in Fig 4B. In the next test, the reference genome was compared to genomes in the same Family or Order. Here, all programs performed less well than in the previous test, with *Mottle* performing the best, followed by *Mash* and then *Swipe*. Again, *Co-Phylog* struggled to unambiguously place genomes correctly at this taxonomic distance. In the final test, the reference genome was compared with ones sharing an Order and Class. This test was far more challenging. Once again, *Mash*, *Mottle* and *BWA-MEM2* were the most effective, showing mainly correct placements with few ambiguous results. The other tools demonstrated high levels of ambiguity in this challenge, being unable to find any regions of homology to calculate a stable distance. *Mottle*, *Mash* and *BWA-MEM2* are therefore useful tools for placing test genomes within known taxonomies, even at large taxonomic distances.

## Conclusions

Pairwise substitution distance has a wide set of applications, from building phylogenies to creating reduced databases. Finding a novel approach in this field can find utilisations in any of these areas, giving opportunities for further refinement specific to certain applications. *Mottle* represents an additional tool in this endeavour. It performs as well as existing programs in correctly identifying taxonomic relationships but comes with the added advantage of provided an accurate numerical estimation of genomic distance over a greater sequence divergence. A limitation of its use, though, is a reduced numerical accuracy, compared to alternative approaches, at low divergences on genomes that behave similarly to a simple substitution model, i.e., where insertions and deletions are uncommon. *Mottle* may therefore be of particular interest to the study of viruses, viral relationships, and viral discovery platforms, where available sequences for reference may be sparse, sequence diversity is high, and insertions/deletions occur at a high rate. The algorithms behind *Mottle* can be applied using any Short-Read mapper, Gradient-Boosted Decision Tree generator, Gradient-Descent software, and Nearest-Neighbour finder. This means that any advancements in such software would give increased efficiency or accuracy to new implementations. Further extensions to the algorithm could include support for amino acid input, simultaneous multiple sequence distance calculation and isolating sub-alignments of continuous homology. In conclusion, *Mottle* is an invaluable, novel approach in substitution distance estimation, with significant performance benefits compared to other algorithms.
